# A Comparative Analysis of Genetic Differentiation across Six Shared Willow Host Species in Leaf- and Bud-Galling Sawflies

**DOI:** 10.1371/journal.pone.0116286

**Published:** 2014-12-31

**Authors:** Sanna A. Leppänen, Tobias Malm, Kaisa Värri, Tommi Nyman

**Affiliations:** 1 Department of Biology, University of Eastern Finland, P. O. Box 111, FI-80101, Joensuu, Finland; 2 Department of Zoology, Swedish Museum of Natural History, P. O. Box 50007, SE-10405, Stockholm, Sweden; University of Arkansas, United States of America

## Abstract

Genetic divergence and speciation in plant-feeding insects could be driven by contrasting selection pressures imposed by different plant species and taxa. While numerous examples of host-associated differentiation (HAD) have been found, the overall importance of HAD in insect diversification remains unclear, as few studies have investigated its frequency in relation to all speciation events. One promising way to infer the prevalence and repeatability of HAD is to estimate genetic differentiation in multiple insect taxa that use the same set of hosts. To this end, we measured and compared variation in mitochondrial COI and nuclear ITS2 sequences in population samples of leaf-galling *Pontania* and bud-galling *Euura* sawflies (Hymenoptera: Tenthredinidae) collected from six *Salix* species in two replicate locations in northern Fennoscandia. We found evidence of frequent HAD in both species complexes, as individuals from the same willow species tended to cluster together on both mitochondrial and nuclear phylogenetic trees. Although few fixed differences among the putative species were found, hierarchical AMOVAs showed that most of the genetic variation in the samples was explained by host species rather than by sampling location. Nevertheless, the levels of HAD measured across specific pairs of host species were not correlated in the two focal galler groups. Hence, our results support the hypothesis of HAD as a central force in herbivore speciation, but also indicate that evolutionary trajectories are only weakly repeatable even in temporally overlapping radiations of related insect taxa.

## Introduction

The extreme species richness of plant-feeding insects has puzzled researchers for decades [1]–[3]. One possible driver of insect herbivore diversification is that shifts onto novel host plants lead to the formation of new species [4]–[6]. This could occur if chemical, ecological, morphological, and phenological differences among plant taxa act as a source of divergent natural selection [7]–[9]. In such cases, host-associated differentiation (HAD) in insect populations can lead to the formation of reproductively partially isolated host races [10], [11] that eventually split into fully separated lineages, each of which is associated with a different plant [12], [13].

Genetic studies have revealed numerous cases of HAD among insect populations and species [3], [10], [14]. However, it is still unknown whether HAD constitutes a generally important driver of herbivore diversification, because this would require knowledge on its frequency in relation to all speciation events [15]–[17]. One way to infer the prevalence of HAD is to estimate genetic divergence in multiple insect taxa that share the same set of host plants: if HAD is common, it should be observable in many of the focal groups [12]. Further, if the buildup of reproductive isolation is predictable, the level of HAD across specific host species and taxa should be roughly equal in independently evolving insect lineages, especially if they are of similar age. Unfortunately, few studies have investigated HAD in more than one herbivore–host taxon pair, meaning that while the literature abounds with convincing case studies, an understanding of the frequency and repeatability of HAD is still lacking [12], [18], [19].

An exceptionally suitable model system for studying parallel HAD is provided by the species-rich network formed by willows (Salicaceae: *Salix* spp.) and willow-galling sawflies that belong to the nematine subtribe Euurina (Hymenoptera: Tenthredinidae). At approximately 300–500 species, willows constitute one of the most diverse plant genera in the Holarctic region [20]–[22]. The circa 400 species of Euurina gallers induce leaf folds or rolls, or various types of closed galls on leaves, petioles, buds, or shoots of *Salix* species (only a handful of leaf-rolling/folding species occur on *Populus* in the same plant family) [23], [24]. Phylogeny-based studies have revealed that gall-type shifts have been relatively rare during the evolutionary history of the Euurina, while the use of *Salix* species has been far more labile [25], [26]. Therefore, extant Euurina species represent the end product of multiple, temporally overlapping radiations of lineages representing different gall morphologies. Another consequence is that willows and Euurina gallers occur in multispecies communities, in which each willow species typically is attacked by numerous sawfly species that induce different galls. Based on subtle morphological differences, most Euurina species are presumed to be strictly monophagous on single *Salix* species [23], [24], but the proposition has rarely been tested using population-genetic methods [27]. As a result, the level of reproductive isolation – and resultant genetic divergence – among the putative species remains largely unstudied.

**Figure 1:**
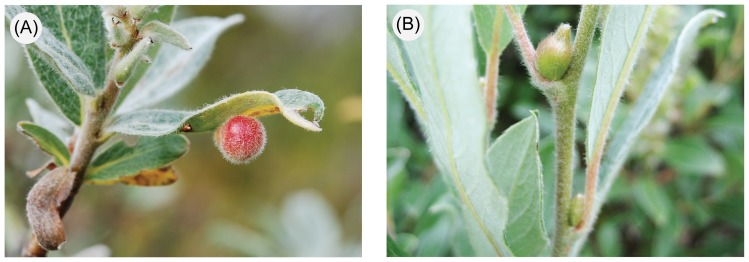
Examples of galls induced by the focal sawfly groups on willows. (A) *Pontania* leaf gall on *Salix glauca*, and (B) *Euura* bud gall on the same host species (upper bud galled, lower one uninfected).

Our present study focused on leaf- and bud-galling sawflies belonging to the *Pontania viminalis*-group and the *Euura mucronata*-group, respectively [23], [24], [28] (Fig. 1). These two taxa often co-occur on sympatric *Salix* species and, therefore, can be considered independent replicates of differentiation and radiation across the same ‘selective background’ formed by willows. Both groups are composed of univoltine closely related species that in many cases are very difficult to separate on the basis of morphological traits [24], [26], [29]. Within Europe, Kopelke [23], [28] recognized 19 host-specific species in the *P. viminalis*-group and 16 species in the *E. mucronata*-group. Kopelke's hypothesis of a strict 1∶1 relationship between willows and various groups of Euurina gallers has, however, been genetically investigated only in northern European *Euura* bud gallers, in which Nyman [27], using allozyme-electrophoretic data, found evidence for the existence of three or four variably specialized lineages across six willow host species. However, the degree of genetic differentiation and reproductive isolation among the lineages remained unclear, and comparable data on the leaf-galling *P. viminalis*-group are still lacking.

In order to study the repeatability of HAD in shared ecological settings, we collected *Pontania* and *Euura* individuals from galls on six willow species that are used by both taxa at two replicate locations in northern Fennoscandia (Abisko in Sweden and Kilpisjärvi in Finland), and then used DNA sequence data from the mitochondrial COI gene and the nuclear ITS2 region to infer their levels of genetic differentiation across host species and geographic locations. Specifically, we wanted to find out: (1) is there evidence for HAD in the two focal species complexes, and (2) is the level of HAD across pairs of willow host species similar in bud- and leaf-galling sawflies?

## Methods

### Sample collection

*Pontania* and *Euura* samples were collected from the surroundings of the Kilpisjärvi research station (69°03′N, 20°48′E) in Finland and the Abisko research station (68°21′N, 18°49′E) in Sweden. Both sites are located *c*. 250 km north of the Arctic Circle, and the distance between them is about 110 km. The stations are surrounded by very similar habitats, with lowlands dominated by mountain birch (*Betula pubescens* ssp. *czerepanovii* (N. I. Orlova) Hämet-Ahti) forests, and a subalpine zone consisting of *Salix* thickets surrounding the tree- and shrubless higher areas and fjell tops. The focal taxa are very common, and no specific permissions were required for collecting samples.

Galler population samples from specific willow species were collected haphazardly and at intervals of at least 20 meters, in order to avoid sampling of closely related individuals. *Euura* bud galls were collected in 1998 from six willow species that occur in both areas: *Salix lanata* L., *S. glauca* L., *S. lapponum* L., *S. phylicifolia* L., *S. myrsinifolia* Salisb., and *S. hastata* L. (Table 1). The larvae were reared to adults and sexed, and then used in the allozyme-electrophoretic study by Nyman [27]. The remains of the individuals were stored frozen in 1.5-ml Eppendorf tubes at −80°C until DNA extraction and sequencing. Only *Euura* males were used in this study: because hymenopteran males are haploid, nuclear sequences could be determined to the haplotype level. *Pontania* larvae were collected in 1997 and 1998 from the aforementioned six willow species, but also from *S. borealis* (Fr.) Nasarov and *S. reticulata* L. In addition, *Pontania* samples were collected from *S. myrsinites* L. in Kilpisjärvi, but leaf galls were absent from this host in Abisko. *Pontania* specimens were stored in 99.5% ethanol at −20°C, but the sex of the larvae could not be determined.

**Table 1:** Numbers of mitochondrial COI gene and nuclear ITS2 region sequences obtained for leaf-galling *Pontania* and bud-galling *Euura* individuals originating from different willow host species and collection sites.

	Kilpisjärvi		Abisko	
*Pontania*	COI	ITS2	COI	ITS2
*Salix lapponum*	9(1)	11	5	5
*Salix hastata*	10(1)	10	9	7
*Salix phylicifolia*	8(1)	6	5(1)	5
*Salix lanata*	10	8	6	6
*Salix glauca*	10(2)	7	8	4
*Salix myrsinifolia*	7	10	5	5
*Salix borealis*	10(1)	8	2	2
*Salix reticulata*	9(5)	8	4(1)	5
*Salix myrsinites*	10	8	0	0
***Euura***				
*Salix lapponum*	10	10	10	10
*Salix hastata*	8(1)	8	10(5)	10
*Salix phylicifolia*	9	9	10	10
*Salix lanata*	10	9	10	10
*Salix glauca*	10	10	9	10
*Salix myrsinifolia*	7	6	7	6

Numbers of heteroplasmic COI sequences are given in parentheses.

We aimed at collecting and sequencing ten sawfly individuals per host species from both locations, but for some of the hosts or sites the final sample sizes were lower due to rarity of galls and/or problems in PCR amplification (Table 1). The total number of sequenced individuals was 245.

### DNA extraction, PCR amplification, sequencing, and sequence alignment

Total genomic DNA was extracted from the samples using DNeasy Tissue Kits (Qiagen, Valencia, CA). A portion of the mitochondrial Cytochrome oxidase I (COI) gene was amplified and sequenced using methods described earlier [30]. In addition, we sequenced a fragment of the nuclear Internal transcribed spacer 2 (ITS2) region, which was amplified using primers AM1 (5′ TGT GAA CTG CAG GAC ACA TGA 3′) and AM2 (5′ ATG CTT AAA TTT AGG GGG TAG TC 3′) [31]. ITS2 amplification and sequencing was done following the same protocols as for COI [30]. The PCR program for ITS2 consisted of an initial denaturing step at 94°C for 5 min, followed by 35 cycles of 1 min denaturing at 94°C, 1 min annealing at 52°C, and 1 min extension at 72°C. We also designed four new internal primers that were used when needed to confirm the sequences of the PCR products: AMfor180 (5′CGA TCA AAG ACT TGT ACA CG 3′), AMfor250 (5′ GTC GCG TCC CAG TGC TAT CTG 3′), AMrev430 (5′ACG AGG ATA AAT TCG ACT ACC 3′), and AMrev460 (5′ GAC GGT CGG ACG TGT ACG T 3′).

The 238 COI sequences were all 810 bp in length, and were read, edited, and aligned using Sequencher version 4.8 (Gene Codes Corp., Ann Arbor, MI). Thirteen cases of heteroplasmic COI sequences were observed in *Pontania* and six in *Euura* (Table 1); we note that since no stop codons were present in the sequences, these instances are more likely to reflect true intra-individual heteroplasmy rather than the presence of nuclear pseudogenes (*cf.* [32]), so the sequences were retained in our analyses. ITS2 sequences of 222 individuals were aligned by implementing ClustalW [33] in Mesquite version 2.74 [34], followed by minor manual correction; the final alignment is 611 bp long. All sequences have been deposited in GenBank under accession numbers KJ155839–KJ156298.

### Phylogeny reconstruction

The COI, ITS2, and combined COI+ITS2 datasets were analyzed in a Bayesian phylogenetic framework in MrBayes version 3.2.2 [35] on the CIPRES Science Gateway [36]. Specimens lacking data for either one of the genes were excluded from the combined-data analysis, making the final number of individuals in the COI+ITS2 dataset 215. Because the ITS2 alignment contained gaps, which are treated as unknowns by model-based methods of phylogenetic inference, gaps were recoded to binary presence/absence characters following the method of Simmons and Ochoterena [37] as implemented in FastGap version 1.2 [38]. In all analyses, we used a mixed substitution model with gamma-categorized rate variation for nucleotide sites, while the recoded gaps were treated as a separate partition of variable, gamma-corrected restriction sites. Ratepr, statefreqpr, shape, revmat, and tratio were left as default and unlinked across partitions. Analyses were run over four chains for 10 million generations while sampling trees every 1,000 generations; run convergence was assessed in Tracer v.1.6.0 [39] to ensure that the 25% burn-in cutoffs included all pre-convergence samples. All trees were midpoint-rooted, following results by [25] and [26].

### Analyses of genetic differentiation

ARLEQUIN version 3.5 [40] was used to estimate pairwise *Φ*_ST_ distances among population samples collected from different hosts and locations, as well as to conduct hierarchical analyses of molecular variance (AMOVA) [41]. Calculations were performed separately for the mitochondrial and nuclear sequence datasets. The best-fitting substitution model for COI ( = TrN+I = TN93+I) was determined under the corrected Akaike Information Criterion (AICc) in jModelTest version 2.1.3 [42]. Because a proportion of invariant sites cannot be included in analyses in ARLEQUIN, we instead used the TN93 substitution model with a gamma correction for rate heterogeneity (α = 0.011, estimated by jModelTest). Analyses of the ITS2 data were based on numbers of pairwise differences among sequences, and gaps were weighted equally to substitutions. As our main aim was to compare levels of HAD in *Euura* and *Pontania* gallers, we included in these analyses only samples originating from the six willow host species that are shared by both groups. Hence, *Pontania* individuals sampled from *S. myrsinites* and *S. reticulata* were excluded, and *S. borealis*-associated *Pontania* larvae were united under *S. myrsinifolia*; *S. borealis* can be considered a subspecies of the latter (*S. myrsinifolia* ssp. *borealis* (Fr.) Hyl.; see [43]), and the population samples collected from these two willows did not differ when samples were pooled across locations (COI: *Φ*_ST_ = −0.033, *P* = 0.472; ITS2: *Φ*_ST_ = 0.000, *P* = 1.000). Hierarchical AMOVAs for genetic differentiation across the population samples were performed using two different grouping hierarchies: collection locations within host species, and *vice versa*. Statistical significance of estimated parameters was evaluated using 10,000 permutations.

Differences in average *Φ*_ST_ between *Pontania* and *Euura* were analyzed with paired-samples *t*-tests in IBM SPSS version 19.0 (IBM SPSS statistics Inc., Chicago, IL, USA). In order to compare *Φ*_ST_ values across host-species pairs, populations sharing hosts in different locations were pooled, and the existence of a correlation between estimates based on the *Pontania* and *Euura* datasets was assessed using a Mantel test employing 10,000 randomizations in PC-ORD version 5.0 [44]. Correlations between among-host differentiation in COI and ITS2 within the two genera were tested similarly.

## Results

### Phylogenetic trees

**Figure 2:**
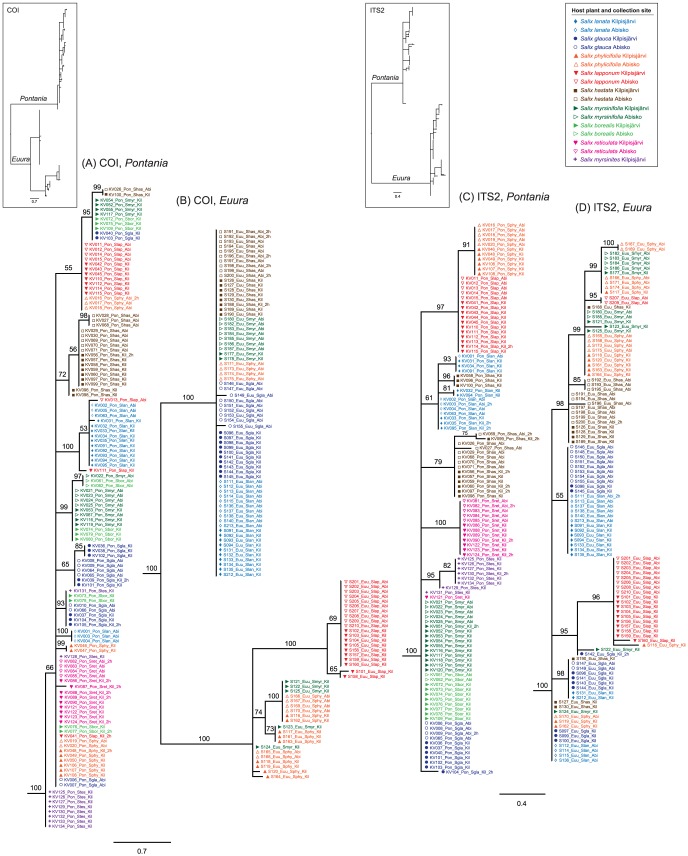
Bayesian phylogenetic trees for mitochondrial COI sequences in (A) *Pontania* and (B) *Euura*, and for nuclear ITS2 sequences in (C) *Pontania* and (D) *Euura*. Outlines of full midpoint-rooted trees for both genes are shown in the insets, host plants are indicated by individual label colours and symbols, and collection sites by symbol fills (see legend). Numbers above branches are posterior probabilities (%), the suffix ‘2 h’ denotes individuals with heteroplasmic COI or heterozygous ITS2 sequences.

Midpoint-rooted 50% majority-rule Bayesian trees show for both COI and ITS2 a highly supported split between *Pontania* and *Euura*, and long branches leading to each genus (insets in Fig. 2). By contrast, genetic distances within the genera are very short, and many groupings therefore remain unresolved or receive weak support (*i.e.*, posterior probability <90%). Nevertheless, all single-gene trees demonstrate clear non-random clustering according to host plant in both focal species complexes.

In the *Pontania* branch of the COI tree (Fig. 2A), no monophyletic groups are formed exclusively by all specimens collected from a single willow host species. However, many clusters consisting of identical or near-identical sequences are dominated by samples from one host species. The *Euura* COI branch (Fig. 2B) shows less resolution, but all individuals collected from *S. lapponum* are grouped within a single clade. Larvae collected from *S. phylicifolia* and *S. myrsinifolia* are found in several groups next to the ‘*S. lapponum* clade,’ but these hosts are also represented in the large, tight clade containing all specimens from *S. lanata*, *S. hastata*, and *S. glauca*.

The *Pontania* half of the ITS2 tree (Fig. 2C) exhibits somewhat clearer host-based grouping than does the corresponding part of the COI tree. Individuals from *S. lapponum* and *S. phylicifolia* form distinct groups, and marked host-based frequency differences are found also in the other clades of the tree, although most specimens collected from *S. myrsinifolia*, *S. borealis*, and *S. glauca* share a single sequence type. Likewise, the *Euura* branch of the ITS2 tree (Fig. 2D) is more structured than the *Euura* COI phylogeny. Individuals from *S. lapponum* form a strongly supported and near-exclusive clade. Notably, individuals from *S. lanata* and *S. glauca* occur in many places across the tree, but are often intermixed in the same clades, and a similar pattern is discernible for samples collected from *S. myrsinifolia* and *S. phylicifolia*.

**Figure 3:**
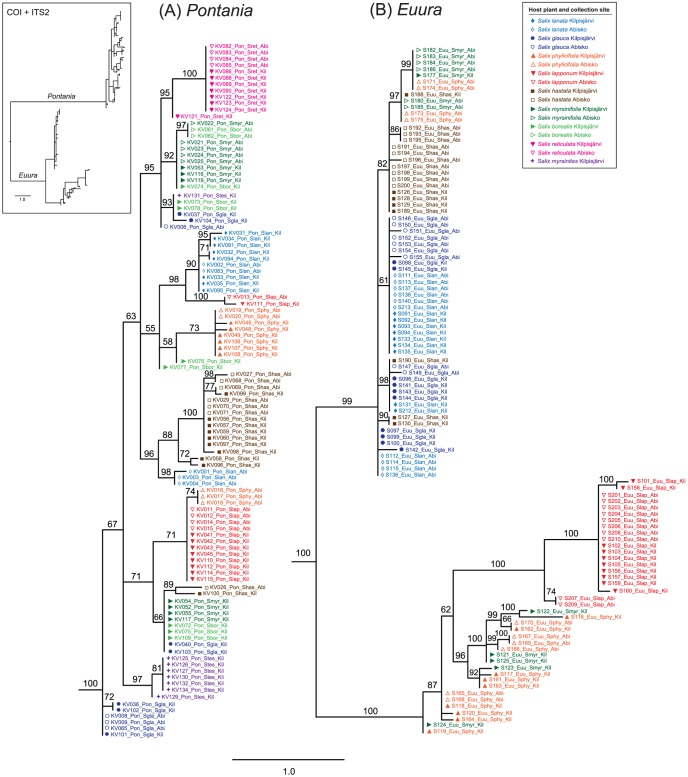
Bayesian phylogenetic tree for combined COI and ITS2 sequence data for (A) *Pontania* and (B) *Euura*. Outline of full midpoint-rooted tree is shown in the inset, host plants are indicated by individual label colours and symbols, and collection sites by symbol fills (see legend). Numbers above branches are posterior probabilities (%).

The overall structure of the combined-data tree (inset in Fig. 3) closely resembles the results of the separate analyses of the COI and ITS2 datasets. The *Pontania* half of the tree (Fig. 3A) groups individuals according to host plants clearer than do the single-gene trees. Individuals originating from *S. reticulata* form a single clade, and individuals from *S. phylicifolia*, *S. lanata*, *S. hastata*, and *S. lapponum* occur in two separate, yet exclusive groups each. Specimens collected from *S. myrsinifolia* and *S. borealis* tend to co-occur in clades, while samples from *S. glauca* are scattered in many clades across the tree. Within *Euura* (Fig. 3B), the combined-data tree largely reflects the COI partition of the dataset, yet provides added resolution as compared to the COI tree. All *S. lapponum*-associated individuals are included in a single monophyletic group. Individuals collected from *S. lanata* and *S. glauca* are intermixed in several shared groups. *S. hastata*-associated specimens occur in several groups, but nearly all of them have very similar overall sequences. Samples from *S. phylicifolia* and *S. myrsinifolia* are intermixed and found in two relatively distant branches on the tree, which seems to be driven by their occurrence in two clades on the COI tree.

### Determinants of population-genetic structure

Within *Pontania*, pairwise COI *Φ*_ST_ values across population samples collected from different locations and hosts ranged from −0.187 to 0.987, and differentiation was statistically significantly non-zero (at *P*<0.05) for 83.33% of the compared pairs (S1 Table). ITS2-based *Pontania Φ*_ST_ values ranged from −0.098 to 1.000, and 84.84% of the contrasts were statistically significant (S2 Table). These results are mirrored in the *Euura* population samples (S1 and S2 Tables), in which pairwise COI *Φ*_ST_ = −0.277–1.000 and ITS2 *Φ*_ST_ = −0.116–0.959 (65.15% and 83.33% of pairs statistically significantly non-zero for COI and ITS2, respectively).

The hierarchical AMOVAs (Tables 2 and 3) show that, regardless of the order of the hierarchy, most of the observed genetic variation in both *Pontania* sequence datasets is explained by the host plant (COI: 50.47–68.66%; ITS2: 87.27–105.91%), while the effect of location is far weaker (COI: −11.10–11.05%; ITS2: −18.21–2.32%). The effect of host species is statistically significant in all cases, whereas the location effect is significantly non-zero only for the COI analysis in which locations are grouped within hosts (Table 2).

**Table 2:** Hierarchical analyses of molecular variance in *Pontania* and *Euura* gallers based on mitochondrial COI gene sequence data.

		*Pontania*			*Euura*		
Hierarchy	Source of variation	Percentage of variation	*Φ*-statistics	*P*-value	Percentage of variation	*Φ*-statistics	*P*-value
Sites within willow hosts	Among hosts	50.47	*Φ*_CT_ = 0.505	<0.001	67.38	*Φ*_CT_ = 0.674	0.033
	Among sites within hosts	11.05	*Φ*_SC_ = 0.223	0.002	16.72	*Φ*_SC_ = 0.512	0.002
	Within sites	38.48	*Φ*_ST_ = 0.615	<0.001	15.91	*Φ*_ST_ = 0.841	<0.001
Willow hosts within sites	Among sites	−11.10	*Φ*_CT_ = −0.111	0.891	−9.75	*Φ*_CT_ = −0.098	0.543
	Among hosts within sites	68.66	*Φ*_SC_ = 0.618	<0.001	92.04	*Φ*_SC_ = 0.839	<0.001
	Within hosts	42.44	*Φ*_ST_ = 0.576	<0.001	17.71	*Φ*_ST_ = 0.823	<0.001

**Table 3:** Hierarchical analyses of molecular variance in *Pontania* and *Euura* gallers based on nuclear ITS2 region sequence data.

		*Pontania*			*Euura*		
Hierarchy	Source of variation	Percentage of variation	*Φ*-statistics	*P*-value	Percentage of variation	*Φ*-statistics	*P*-value
Sites within willow hosts	Among hosts	87.27	*Φ*_CT_ = 0.873	<0.001	61.63	*Φ*_CT_ = 0.616	<0.001
	Among sites within hosts	2.32	*Φ*_SC_ = 0.182	0.085	5.28	*Φ*_SC_ = 0.138	<0.001
	Within sites	10.40	*Φ*_ST_ = 0.896	<0.001	33.09	*Φ*_ST_ = 0.669	<0.001
Willow hosts within sites	Among sites	−18.21	*Φ*_CT_ = −0.182	0.918	−9.59	*Φ*_CT_ = −0.096	0.751
	Among hosts within sites	105.91	*Φ*_SC_ = 0.896	<0.001	72.96	*Φ*_SC_ = 0.666	<0.001
	Within hosts	12.30	*Φ*_ST_ = 0.877	<0.001	36.63	*Φ*_ST_ = 0.634	<0.001

In analogous analyses based on the *Euura* samples, most of the observed genetic variation is again explained by host species (COI: 67.38–92.04%; ITS2: 61.63–72.96%; all *P*<0.05), while the influence of location is weak (COI: −9.75–16.72%; ITS2: −9.59–5.28%) and statistically significantly different from zero only when location is nested within host in the hierarchy (Tables 2 and 3).

### Comparisons of differentiation in *Pontania* and *Euura*

In the COI dataset, average differentiation across hosts and locations (S1 Table) was statistically indistinguishable in the *Euura* (*Φ*_ST_ = 0.547±0.052 [mean ± s.e. mean]) and *Pontania* (*Φ*_ST_ = 0.538±0.035) samples (paired-samples *t*-test; *t*_65_ = −0.170, *P* = 0.865). However, for the ITS2 data (S2 Table), mean differentiation was significantly higher within *Pontania* (*Φ*_ST_ = 0.757±0.042) than in *Euura* (*Φ*_ST_ = 0.516±0.036) (paired-samples *t*-test; *t*_65_ = 5.359, *P*<0.001).

**Figure 4:**
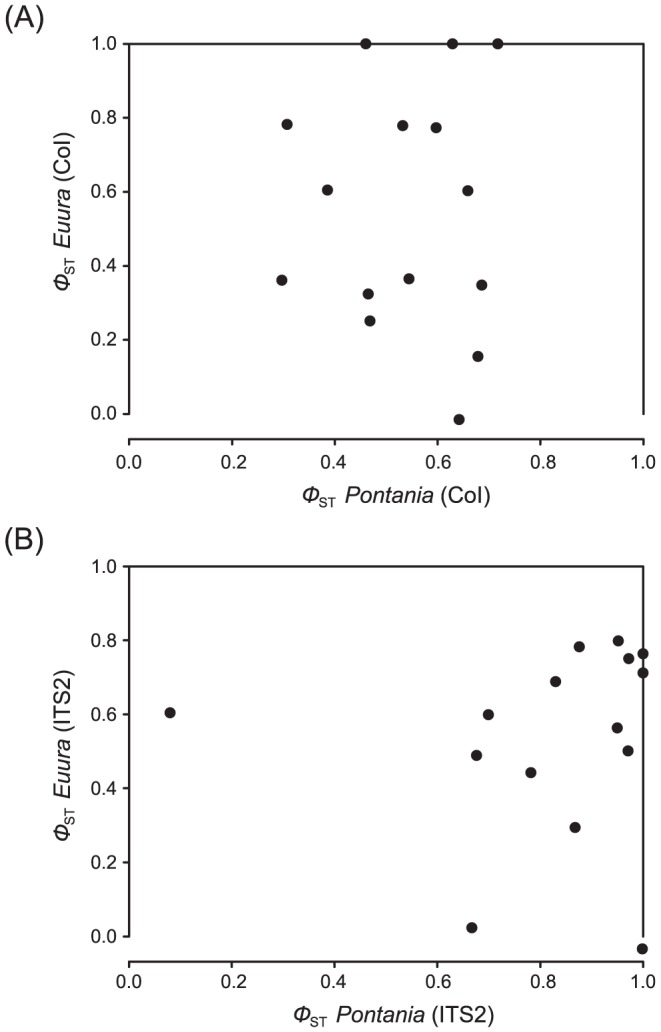
Scatterplots of pairwise *Φ*_ST_ values estimated across pairs of willow host species in *Pontania* and *Euura* according to (A) COI and (B) ITS2 sequence data.

When populations from conspecific hosts in different locations were pooled, average COI differentiation across host species (Fig. 4A) did not differ between *Euura* (*Φ*_ST_ = 0.555±0.085) and *Pontania* (*Φ*_ST_ = 0.538±0.035) (paired-samples *t*-test; *t*_14_ = −0.186, *P* = 0.855). By contrast, mean differentiation in the ITS2 dataset (Fig. 4B) was significantly higher in *Pontania* (*Φ*_ST_ = 0.822±0.061) than in *Euura* (*Φ*_ST_ = 0.531±0.067) (paired-samples *t*-test; *t*_14_ = 3.321, *P* = 0.005).

Comparisons of *Φ*_ST_ values estimated across corresponding pairs of willow host species in the *Euura* and *Pontania* samples (Fig. 4) showed no evidence of a correlation in either the COI (Mantel test statistic *r* = −0.175, *P* = 0.256) or the ITS2 (*r* = 0.080, *P* = 0.393) datasets. Differentiation across hosts in the COI and ITS2 datasets correlated statistically significantly within *Euura* (Mantel test statistic *r* = 0.559, *P* = 0.022) but not within *Pontania* (Mantel test statistic *r* = 0.216, *P* = 0.209).

## Discussion

One of the most central questions in evolutionary research concerns the repeatability of evolution [45]: if independent organismal lineages were to start from exactly the same conditions in identical ecological settings, would their evolutionary trajectories be similar? In research on replicated evolution, the main advantages of plant-feeding insects are their extreme species diversity and the fact that defining available niches (hosts) is easy [46]. For practical reasons, analyses of HAD and ecologically-based speciation in herbivores have thus far largely involved single insect taxa and their host plants (*e.g.*, [7], [13], [47], [48]), but two recent studies have emphasized the need for broader analyses that search for commonalities across multiple herbivore groups: Stireman *et al.* [12] found evidence of HAD in four out of nine insect taxa that attack the North American goldenrod species *Solidago altissima* and *S. gigantea*, and Dickey and Medina [18] demonstrated that while the aphid *Monelliopsis pecanis* exhibits HAD across two broadly sympatric hickory species (*Carya aquatica* and *C. illinoinensis*), genetic structuring is essentially absent in the co-occurring tortricid moth *Gretchena bolliana*. Following the same logic, but on a larger scale, we estimated and compared the levels of HAD in two species-rich and closely related sawfly species complexes that exhibit wide overlap in their collective host repertoires. This approach makes it possible to infer how common HAD is and, more importantly, to study the reproducibility of genetic differentiation in similar environments.

### Effects of geography and host-plant species on genetic differentiation in *Pontania* and *Euura* gallers

In addition to HAD, genetic divergence and speciation in insects could be driven by simple geographic isolation [17], [49]. An illuminating example in this respect is provided by the study by Craft *et al.* [50], who investigated DNA barcode variation in 28 lepidopteran (presumed) species collected from four plant families in eight locations scattered across the northern lowlands of New Guinea. They found substantial population subdivision in roughly half of the studied taxa: 14% showed evidence of HAD, 18% exhibited an isolation-by-distance pattern, 21% were differentiated by both hosts and geography, while the population structures of the remaining 46% were equivocal or essentially panmictic.

In our focal system, hierarchical AMOVAs (Tables 2 and 3) and pairwise *Φ*_ST_ estimates (S1 and S2 Tables) demonstrated frequent geographically-based population subdivision within both genera. However, the AMOVAs also showed that most of the variation in the mitochondrial and nuclear sequence datasets is explained by host species rather than by sampling locality; in the phylogenetic trees (Figs. 2 and 3), this is seen in that specimens tend to cluster according to willow species instead of collection site. Two locations were included in our analyses mainly in order to obtain independent replicate population samples within the studied taxa, and the relatively weak location effect was somewhat expected because the distance between Kilpisjärvi and Abisko is only slightly over 100 km; while this clearly exceeds the dispersal ability of individual sawflies, the homogeneous intervening landscape and vegetation present few obstacles to gene flow. However, both of the focal species-groups are distributed across the Holarctic region and individual species can have very broad ranges [24], [51], so stronger intraspecific spatial differentiation would be expected on larger geographical scales.

Genetic distances among clusters within the two genera are, however, very small, and most clades are to some degree intermixed with respect to host species, or at least include one or a few individuals from atypical hosts (Figs. 2 and 3). The absence of fixed diagnostic differences among the presumed species, and the lack of a correlation between COI and ITS2 differentiation within *Pontania*, could be explained by stochastic retention of ancestral polymorphisms in recently diverged lineages [52], [53]. In addition to coalescent stochasticity and incomplete lineage sorting, the observed shared polymorphisms and topological conflicts between the COI and ITS2 phylogenies could reflect occasional interbreeding among host races or incipient species that are still incompletely reproductively isolated from each other. Evidence for hybridization, and especially introgression of mtDNA haplotypes across species boundaries, has commonly been found in studies on genetic differentiation in insect herbivores [54]–[57]. Studies employing larger numbers of markers and individuals are needed to estimate the extent of hybridization in our focal species groups, but we note that occasional interbreeding among partly separated galler lineages seems probable in light of the recently-demonstrated widespread occurrence of interspecific gene flow in their *Salix* host plants [22].

Using allozyme-electrophoretic data, Nyman [27] showed that northern Fennoscandian bud-galling *Euura* lineages are divided into at least three mono- or oligophagous species. Our DNA sequence-based analyses support these results, especially with respect to the genetic distinctness of *S. lapponum*-associated samples (*i.e.*, *E. lappo* Malaise), and the similarity of gallers on *S. lanata* and *S. glauca* (Fig. 3B). Individuals collected from the latter two willow species are essentially identical and intermixed in the trees, meaning that, like the earlier allozyme-based analyses, our results contradict Kopelke's [28] suggestion of the presence of two strict specialist species (*E. lanatae* and *E. boreoalpina*, respectively) on these presumedly distantly related [21] but chemically relatively similar [58] hosts. By contrast, our combined dataset supports Kopelke's [28] decision to elevate Malaise's [29] ‘*E. lappo* var. *hastatae*’ to species rank (*i.e.*, *E. hastatae*), although the species cannot be identified using COI data alone (Fig. 2B). The intriguing grouping of individuals collected from *S. phylicifolia* and *S. myrsinifolia* into two separate mixed clusters in the combined-data tree (Fig. 3B) seems to be driven mainly by the mitochondrial sequences (Fig. 2B), so we tentatively suggest that these samples represent a single lineage.

Host-based clustering is equally evident in the leaf-galling *Pontania* complex, in which especially samples collected from *S. myrsinites* and the arctic-alpine dwarf willow *S. reticulata* form distinct clades (Figs. 2 and 3; these clades correspond to the morphologically defined *P. myrsiniticola* Kopelke and *P. reticulatae* Malaise, respectively). The *S. myrsinites*-associated individual KV131 exhibited an atypical sequence in both COI and ITS2; therefore, it most likely indicates an oviposition error resulting in successful gall induction and larval development on a non-host willow. *Pontania* individuals sampled from the remaining *Salix* species are less consistently clustered according to their hosts, but the marked frequency differences among host-based population samples – resulting in statistically highly significant levels of genetic differentiation – indicate the presence of multiple independent lineages. However, our results cast doubt on the existence of separate leaf-galling species on *S. myrsinifolia* and *S. borealis* (*P. varia* and *P. norvegica*, respectively), as suggested by Kopelke [23]. In this respect, our results are in line with the interpretation that, rather than being a distinct species, *S. borealis* is a northern Fennoscandian high-altitude form or subspecies of the more widely distributed *S. myrsinifolia* (*e.g.*, [43]).

### The repeatability of HAD in gall-inducing sawflies

Phylogenetic studies have shown that, while the studied leaf-galling *Pontania* and bud-galling *Euura* groups are not sister taxa, they are of roughly equal age [25], [26]. Hence, it can be argued that the evolutionary opportunities and time available for genetic differentiation and speciation by specialization have been more or less equal in both taxa. Evolutionary repeatability should thus be manifest as comparable average levels of genetic subdivision across the studied hosts, but also as a correlation in the level of HAD across specific pairs of host species.

These predictions are not supported by our results: first, although we found clear evidence for frequent HAD in both galler taxa, average *Φ*_ST_ estimates across host species were similar only in the mitochondrial COI dataset, while differentiation in the nuclear ITS2 data was statistically significantly higher in *Pontania* than in *Euura*. One possible explanation for the discrepancy could be that the *Pontania* group is slightly older than the *Euura* group; if this is the case, our results would resemble the findings of Stireman *et al.* [12], who, in the aforementioned system including two *Solidago* species, found widely differing divergence times in *Gnorimoschema* moths, *Rhopalomyia* gall midges, and *Eurosta* tephritid flies. However, an alternative possibility is that the average rate of genetic divergence has been faster in *Pontania*. Second, lack of evolutionary replicability is indicated by the ‘shotgun’ scatterplots of across-host *Φ*_ST_ estimates in the two focal sawfly genera (Fig. 4). The disparate levels of differentiation suggest that although leaf- and bud-galling sawflies share a common taxonomic resource base, they differ in the way that they experience the adaptive landscape imposed by *Salix* species. This could, for example, be related to differences in the chemistry of leaves or buds, or to differences in the phenology of these tissues during the growing season. Bud gallers exhibit clear interspecific variation in average emergence time after pupation [59], and these differences are most likely linked to the phenology of their *Salix* hosts. In particular, differences in relative phenology across tissue types could lead to differential levels of gene flow among host-adapted insect lineages.

## Conclusions

Studies on evolutionary radiations of taxa experiencing shared ‘adaptive landscapes’ can provide valuable insights into mechanisms that underlie ecological, phenotypic, and genetic divergence and/or convergence [60]–[63]. Our comparative analysis of genetic differentiation across shared *Salix* host species in northern Fennoscandian leaf-galling *Pontania* and bud-galling *Euura* sawflies revealed clear and frequent HAD in both species complexes. Nevertheless, mitochondrial and nuclear sequence variation is in most cases not diagnostic to samples collected from a given willow species, suggesting that, within the groups, species boundaries are still weakly defined, and that the identified lineages are positioned somewhere along the continuum between host races and fully independent species [10], [13], [64]. Like the few prior comparative analyses of HAD in multiple insect herbivore taxa [12], [18], [50] (see also [46]), our results suggest that speciation by ecological specialization plays a central role in herbivore diversification. However, the results have to be interpreted cautiously: like other endophagous insect groups, gall inducers are known to be unusually specialized in their host use [65], [66], meaning that the overall frequency of niche-driven speciation could be lower if other insect feeding guilds are also considered [12].

Despite genetic differentiation being present in both of the focal galler groups, we found differences in the overall level of HAD in the groups, and especially in the differentiation estimated across specific pairs of host species. This indicates that although the ecological selective background has been identical for both taxa, their evolutionary trajectories have been different. While the reasons for the disparities necessarily remain speculative, the few comparative studies done so far suggest that idiosyncrasies across herbivore taxa are likely to be a common feature in multispecies plant–herbivore systems [12], [50]. In this way, our results support Gould's [45] view that evolutionary trajectories are only weakly repeatable during replicated independent radiations.

## Supporting Information

S1 Table **Pairwise** ***Φ*** **_ST_ estimates among** ***Pontania*** **(below diagonal) and** ***Euura*** **(above diagonal) population samples collected from six different willow species in two locations, based on mitochondrial COI gene sequence data.** Willow host names are abbreviated as follows: Smyr = *S. myrsinifolia*, Slap = *S. lapponum*, Sphy = *S. phylicifolia*, Sgla = *S. glauca*, Shas = *S. hastata*, Slan = *S. lanata*, collection sites are indicated by letters in parentheses (A = Abisko, K = Kilpisjärvi). All pairwise estimates are statistically significant at the *P*<0.05 level, except for values in parentheses = n.s.
(DOCX)

S2 Table **Pairwise** ***Φ*** **_ST_ estimates among** ***Pontania*** **(below diagonal) and** ***Euura*** **(above diagonal) population samples collected from six different willow species in two locations, based on nuclear ITS2 region sequence data.** Willow host names are abbreviated as follows: Smyr = *S. myrsinifolia*, Slap = *S. lapponum*, Sphy = *S. phylicifolia*, Sgla = *S. glauca*, Shas = *S. hastata*, Slan = *S. lanata*, collection sites are indicated by letters in parentheses (A = Abisko, K = Kilpisjärvi). All pairwise estimates are statistically significant at the *P*<0.05 level, except for values in parentheses = n.s.
(DOCX)
