# Supplementary material for: A Comparative Analysis of Genetic Differentiation across Six Shared Willow Host Species in Leaf- and Bud-Galling Sawflies
Source: PLoS One. 2014 Dec 31;9(12):e116286. doi: 10.1371/journal.pone.0116286 (PMC4281154; doi:10.1371/journal.pone.0116286)
Supplement: S2 Table — Pairwise Φ ST estimates among Pontania (below diagonal) and Euura (above diagonal) population samples collected from six different willow species in two locations, based on nuclear ITS2 region sequence data. Willow host names are abbreviated as follows: Smyr = S. myrsinifolia, Slap = S. lapponum, Sphy = S. phylicifolia, Sgla = S. glauca, Shas = S. hastata, Slan = S. lanata, collection sites are indicated by letters in parentheses (A = Abisko, K = Kilpisjärvi). All pairwise estimates are statistically significant at the P<0.05 level, except for values in parentheses = n.s. (DOCX) [file pone.0116286.s002.docx]

**Table S2** Pairwise *Φ*_ST_ estimates among *Pontania* (below diagonal) and *Euura* (above diagonal) population samples collected from six different willow species in two locations, based on nuclear ITS2 region sequence data. Willow host names are abbreviated as follows: Smyr = *S. myrsinifolia*, Slap = *S. lapponum*, Sphy = *S. phylicifolia*, Sgla = *S. glauca*, Shas = *S. hastata*, Slan = *S. lanata*, collection sites are indicated by letters in parentheses (A = Abisko, K = Kilpisjärvi). All pairwise estimates are statistically significant at the *P* < 0.05 level, except for values in parentheses = n.s.

|  |  |  |  |  |  |  | *Euura* |  |  |  |  |  |  |
| --- | --- | --- | --- | --- | --- | --- | --- | --- | --- | --- | --- | --- | --- |
|  |  | Smyr (A) | Smyr (K) | Slap (A) | Slap (K) | Sphy (A) | Sphy (K) | Sgla (A) | Sgla (K) | Shas (A) | Shas (K) | Slan (A) | Slan (K) |
|  | Smyr (A) | **–** | (0.152) | 0.699 | 0.959 | (–0.051) | (0.155) | 0.824 | 0.713 | 0.722 | 0.601 | 0.849 | 0.815 |
|  | Smyr (K) | (0.000) | **–** | 0.542 | 0.857 | (0.045) | (–0.098) | 0.587 | 0.424 | 0.432 | 0.211 | 0.568 | 0.565 |
|  | Slap (A) | 1.000 | 1.000 | **–** | (0.105) | 0.674 | 0.479 | 0.683 | 0.593 | 0.692 | 0.610 | 0.678 | 0.669 |
|  | Slap (K) | 1.000 | 1.000 | (0.000) | **–** | 0.901 | 0.770 | 0.925 | 0.865 | 0.938 | 0.905 | 0.942 | 0.922 |
|  | Sphy (A) | 1.000 | 1.000 | 1.000 | 1.000 | **–** | (0.081) | 0.711 | 0.606 | 0.545 | 0.416 | 0.707 | 0.698 |
| *Pontania* | Sphy (K) | 1.000 | 1.000 | 1.000 | 1.000 | (0.000) | **–** | 0.469 | 0.324 | 0.274 | (0.083) | 0.422 | 0.448 |
|  | Sgla (A) | (0.000) | (0.000) | 1.000 | 1.000 | 1.000 | 1.000 | **–** | 0.242 | 0.727 | 0.528 | (0.068) | (–0.116) |
|  | Sgla (K) | (0.000) | (0.149) | 0.936 | 0.959 | 0.946 | 0.950 | (–0.098) | **–** | 0.571 | 0.258 | 0.198 | 0.208 |
|  | Shas (A) | 0.943 | 0.970 | 0.966 | 0.978 | 0.969 | 0.972 | 0.925 | 0.892 | **–** | 0.212 | 0.725 | 0.715 |
|  | Shas (K) | 0.577 | 0.714 | 0.783 | 0.844 | 0.804 | 0.817 | 0.503 | 0.548 | (0.217) | **–** | 0.478 | 0.505 |
|  | Slan (A) | 0.864 | 0.931 | 0.968 | 0.981 | 0.972 | 0.975 | 0.812 | 0.658 | 0.917 | 0.519 | **–** | (0.055) |
|  | Slan (K) | 0.732 | 0.839 | 0.922 | 0.949 | 0.932 | 0.938 | 0.668 | 0.621 | 0.891 | 0.569 | (0.066) | **–** |
